# Antibody conversion rates to SARS-CoV-2 in saliva from children attending summer schools in Barcelona, Spain

**DOI:** 10.1186/s12916-021-02184-1

**Published:** 2021-11-23

**Authors:** Carlota Dobaño, Selena Alonso, Mariona Fernández de Sevilla, Marta Vidal, Alfons Jiménez, Gemma Pons Tomas, Chenjerai Jairoce, María Melé Casas, Rocío Rubio, María Hernández García, Gemma Ruiz-Olalla, Mònica Girona-Alarcón, Diana Barrios, Rebeca Santano, Robert A. Mitchell, Laura Puyol, Leonie Mayer, Jordi Chi, Natalia Rodrigo Melero, Carlo Carolis, Aleix Garcia-Miquel, Elisenda Bonet-Carne, Joana Claverol, Marta Cubells, Claudia Fortuny, Victoria Fumadó, Cristina Jou, Carmen Muñoz-Almagro, Luis Izquierdo, Quique Bassat, Eduard Gratacós, Ruth Aguilar, Juan José García-García, Gemma Moncunill, Iolanda Jordan

**Affiliations:** 1 ISGlobal, Hospital Clínic - Universitat de Barcelona, Barcelona, Catalonia Spain; 2 CIBER de Enfermedades Infecciosas, Madrid, Spain; 3 Consorcio de Investigación Biomédica en Red de Epidemiología y Salud Pública (CIBERESP), Madrid, Spain; 4 Institut de Recerca Sant Joan de Déu, Esplugues, Barcelona, Spain; 5 Pediatrics Department, Hospital Sant Joan de Déu, Universitat de Barcelona, Esplugues, Barcelona, Spain; 6 Paediatric Intensive Care Unit, Hospital Sant Joan de Déu, Universitat de Barcelona, Barcelona, Spain; 7 Biomolecular Screening and Protein Technologies Unit, Centre for Genomic Regulation (CRG), The Barcelona Institute of Science and Technology, Barcelona, Spain; 8 Fetal Medicine Research Center (Hospital Clínic and Hospital Sant Joan de Déu), Universitat de Barcelona, Barcelona, Spain; 9 Institut d’Investigacions Biomèdiques August Pi i Sunyer (IDIBAPS), Barcelona, Spain; 10 Universitat Politècnica de Catalunya, BarcelonaTech, Barcelona, Spain; 11 Fundació Sant Joan de Déu, Barcelona, Spain; 12 Infectious Diseases Department, Hospital Sant Joan de Déu, Barcelona, Spain; 13 Department of Pathology and Biobank Hospital Sant Joan de Déu, Barcelona, Spain; 14 CIBERER, Instituto de Salud Carlos III, Barcelona, Spain; 15 Department of Medicine, Universitat Internacional de Catalunya, Barcelona, Spain; 16 Molecular Microbiology Department, Hospital Sant Joan de Déu, Esplugues, Barcelona, Spain; 17 Centro de Investigação em Saúde de Manhiça (CISM), Maputo, Mozambique; 18 ICREA, Pg. Lluís Companys 23, 08010 Barcelona, Spain; 19 Center for Biomedical Research on Rare Diseases (CIBER-ER), Madrid, Spain

**Keywords:** SARS-CoV-2, Antibody conversion, Saliva, Children, Schools

## Abstract

**Background:**

Surveillance tools to estimate viral transmission dynamics in young populations are essential to guide recommendations for school opening and management during viral epidemics. Ideally, sensitive techniques are required to detect low viral load exposures among asymptomatic children. We aimed to estimate SARS-CoV-2 infection rates in children and adult populations in a school-like environment during the initial COVID-19 pandemic waves using an antibody-based field-deployable and non-invasive approach.

**Methods:**

Saliva antibody conversion defined as ≥ 4-fold increase in IgM, IgA, and/or IgG levels to five SARS-CoV-2 antigens including spike and nucleocapsid constructs was evaluated in 1509 children and 396 adults by high-throughput Luminex assays in samples collected weekly in 22 summer schools and 2 pre-schools in 27 venues in Barcelona, Spain, from June 29th to July 31st, 2020.

**Results:**

Saliva antibody conversion between two visits over a 5-week period was 3.22% (49/1518) or 2.36% if accounting for potentially cross-reactive antibodies, six times higher than the cumulative infection rate (0.53%) assessed by weekly saliva RT-PCR screening. IgG conversion was higher in adults (2.94%, 11/374) than children (1.31%, 15/1144) (*p*=0.035), IgG and IgA levels moderately increased with age, and antibodies were higher in females. Most antibody converters increased both IgG and IgA antibodies but some augmented either IgG or IgA, with a faster decay over time for IgA than IgG. Nucleocapsid rather than spike was the main antigen target. Anti-spike antibodies were significantly higher in individuals not reporting symptoms than symptomatic individuals, suggesting a protective role against COVID-19.

**Conclusion:**

Saliva antibody profiling including three isotypes and multiplexing antigens is a useful and user-friendlier tool for screening pediatric populations to detect low viral load exposures among children, particularly while they are not vaccinated and vulnerable to highly contagious variants, and to recommend public health policies during pandemics.

**Supplementary Information:**

The online version contains supplementary material available at 10.1186/s12916-021-02184-1.

## Background

Children infected with the severe acute respiratory syndrome coronavirus (SARS-CoV-2) usually present milder forms of the coronavirus disease (COVID-19) or are often asymptomatic, although they seem to be similarly susceptible to getting infected and therefore transmit the virus [1–4]. The lack of attention to this age group has prevented evidence-based information to guide public health policies specifically designed for this population. There is a need to have solid data on how COVID-19 affects children and what is their contribution to overall community transmission [4–7], particularly while they are not vaccinated and more contagious viral variants of concern circulate worldwide.

Most clinical and epidemiological studies report that children are diagnosed less often with COVID-19 [1, 8] but still, there are confounding factors and controversial reports [9, 10]. Several hypotheses have been postulated for the milder presentation of COVID-19 in children, including a putative protective role of pre-existing cross-reactive antibodies to common cold human coronaviruses (HCoV) [11, 12], lower expression of angiotensin-converting enzyme 2 (ACE2) [13], and lower pro-inflammatory propensity in their immune system [14].

SARS-CoV-2 diagnostic procedures implemented in children are essentially the same as in adults. Nasopharyngeal swabs for real-time polymerase chain reaction (RT-PCR) or protein antigen diagnosis are the preferred because of their higher sensitivity and specificity [15]. To reduce the inconvenience and discomfort of nasopharyngeal samples, nasal swabs have also been approved [16]. In addition, non-invasive and better-accepted saliva sampling for RT-PCR has shown similar results to nasopharyngeal swabs [17]. However, such methods diagnose current infection but do not establish the percentage of the population that has been exposed to SARS-CoV-2. For this, antibody-based methods are more appropriate, given that certain immunoglobulins persist over time. Furthermore, antibody surveillance could increase the sensitivity to detect incidence of new cases in longitudinal cohorts by assessing antibody conversion rates in prospective samples, particularly among asymptomatic children who may have lower viral loads and possibly more frequent false negatives for RT-PCR and/or for antigen detection tests.

Antibody assays are usually performed using plasma/serum samples and can be done in saliva samples [18–20], although they are not implemented in clinical practice. They offer many logistic advantages over tests requiring blood samples, especially in pediatric patients and large studies. Versatile multiplex antibody assays measuring several isotypes (IgM, IgA, IgG) and multiple SARS-CoV-2 antigens [21] offer the greatest sensitivity to detect and accurately quantify a breadth of specificities, increasing the potential to identify recently and past exposed individuals, even if they have lower antibody levels, e.g., in asymptomatic subjects. In addition, IgA plays a very important role in COVID-19 immunity [22], and interrogating saliva samples can shed more light into mechanisms of mucosal protection.

The objective of this study was to determine SARS-CoV-2 exposure and antibody conversion in two consecutive saliva samples, as a proxy of seroconversion, in children and adult populations in a school-like environment, between the first and second COVID-19 pandemic waves in Spain, using a friendly and convenient SARS-CoV-2 antibody conversion technique.

## Methods

### Design, subjects, and samples

A cohort of 1907 children (age 0–14 years old) attending 22 summer schools and 2 pre-schools, and adult staff working at the same facilities, located in 27 different venues in the Barcelona metropolitan region, Spain, was followed up from June 29th to July 31st, 2020. Symptomatic children were defined as those with acute respiratory infection including fever, cough, headache, gastrointestinal symptoms, rhinorrhea or nasal congestion, anosmia or ageusia, dyspnea, and myalgia.

Saliva samples were collected weekly over a 5-week period with Oracol devices (Malvern, UK) for optimal harvesting of crevicular fluid enriched with serum antibodies [23, 24], centrifuged, heat inactivated (60 °C, 30 min), and frozen until antibody analysis.

### Laboratory measurements

Saliva SARS-CoV-2 RT-PCR detection was performed as described [25]. Levels of IgG, IgA, and IgM against SARS-CoV-2 nucleocapsid (N) full-length (FL) and C-terminus (CT) [26], spike (S), S2, and RBD proteins, were measured by Luminex assays [21] (details in Additional file 1: Detailed methods) in saliva samples diluted 1:10 in 384-well plates, with paired samples from the same individual run together. Pre-pandemic negative controls were not available. Samples were acquired on a Flexmap 3D xMAP® and median fluorescent intensities (MFI) were exported for each analyte using xPONENT.

### Statistical data analysis

Non-parametric Mann-Whitney *U* tests were used in boxplots to compare levels (log_10_MFI) of each antibody/antigen pair between study groups. Radar plots were used to compare median MFIs of antibody responses between study groups by Mann-Whitney *U* test, adjusting *p* values for multiple comparison by Benjamini-Hochberg. Heatmaps with hierarchical clustering (by Euclidian or Canberra methods) were used to evaluate patterns of responses at the individual level. To quantify how many participants got infected during the study period, we considered at least a 3–4-fold increase (FC) in antibody levels between two consecutive visits [19]. To define the saliva antibody conversion rate, we applied the more stringent threshold of ≥ 4-FC in antibody levels from the first to the last week visit only in the subset of individuals in whom at least two samples were collected ≥ 6 days apart. Saliva antibody reversion was defined as a ≥4-FC decrease in antibody levels from the first to the last week visit in the same subset of individuals (see Additional file 1: Detailed methods).

Saliva antibody conversion rates were finally compared depending on the age, sex, and the presence or not of symptoms.

## Results

The characteristics of the participants tested for saliva antibodies are summarized in Table 1. Detailed baseline characteristics and incidence of RT-PCR infections in the cohort were reported elsewhere [25] and in Additional file 2: Table S1. We processed 5368 saliva samples from 1497 children and 410 adults. Only 5 adults had a COVID-19 prior diagnosis. For the antibody determinations, 3475 inactivated saliva samples with sufficient volume, including first and last paired visits (1568 samples each) and those with only one visit available (339 samples), were analyzed. Median time between first and last visits, excluding single visits, was 14 days (SD 6.44, IQR 7–21). Between those two visits, 7 children and 3 adults tested positive for SARS-CoV-2 RT-PCR.

**Table 1:** Characteristics of participants in the summer school longitudinal study. All individuals with at least one saliva sample of sufficient volume available are included

	Initial visit	Final visit	Single visit
	(*N*=1568)	(*N*=1568)	(*N*=339)
Age continuous median^a^	8.0 (5.0-14.0)		7.0 (5.0-10.0)
Age stratified^b^			
Children	1181 (75.3%)		316 (93.2%)
Adults	387 (24.7%)		23 (6.8%)
Sex			
Male	756 (48.2%)		184 (54.3%)
Female	812 (51.8%)		155 (45.7%)
Dates collection^c^			
1^st^ week	465 (29.7%)	0 (0.0%)	59 (17.4%)
2^nd^ week	873 (55.7%)	46 (2.9%)	175 (51.3%)
3^rd^ week	216 (13.8%)	385 (24.6%)	73 (21.5%)
4^th^ week	14 (0.9%)	603 (38.5%)	25 (7.4%)
5^th^ week	0 (0.0%)	535 (34.1%)	8 (2.4%)
SARS-CoV-2 RT-PCR^d^			
Positive	2 (0.1%)	7 (0.4%)	2 (0.6%)
Negative	1552 (99.2%)	1557 (99.5%)	335 (98.8%)
Indeterminate	1 (0.1%)	1 (0.1%)	0 (0.0%)
Not valid	9 (0.6%)	0 (0.0%)	2 (0.6%)
Symptoms			
Yes	71 (4.5%)	76 (4.8%)	16 (4.7%)
Children	62 (5.2%)	65 (5.5%)	16 (5.1%)
Adults	9 (2.3%)	11 (2.8%)	0 (0.0%)
No	1492 (95.2%)	1492 (95.2%)	323 (95.3%)
Children	1119 (94.8%)	1116 (94.5%)	300 (94.9%)
Adults	378 (97.7%)	376 (97.2%)	23 (100%)

^a^Missing age of 9 adults. IQR in parenthesis

^b^Adult: Age 15 years or older

^c^Average (median) time in days between initial and final visit was 14 (SD 6.69, IQR 7.0–21.0); 15.69 (SD 6.44, IQR 10–21) if only those with ≥ 6 days between visits are included

^d^In the saliva antibody study cohort, there were 7 children and 3 adults who tested RT-PCR positive. Among them, one child and one adult were single visits. There was one adult who was positive at the first visit and negative at the last visit, and one child who was positive at both visits; therefore there were 11 positive samples. Among the paired samples, there were 8 with a positive RT-PCR at any timepoint (6 children, 2 adults). In the main infection cohort study [25], 12 participants (9 children among them) were positive by RT-PCR at least in one visit, and from 11 of them there was a saliva sample available for serology. 3446 samples were RT-PCR negative, 2 indeterminate, 11 invalid, and 7 non-available. Only 5 adults have had COVID-19 before the study

### SARS-CoV-2 antibody saliva conversion

**Fig. 1:**
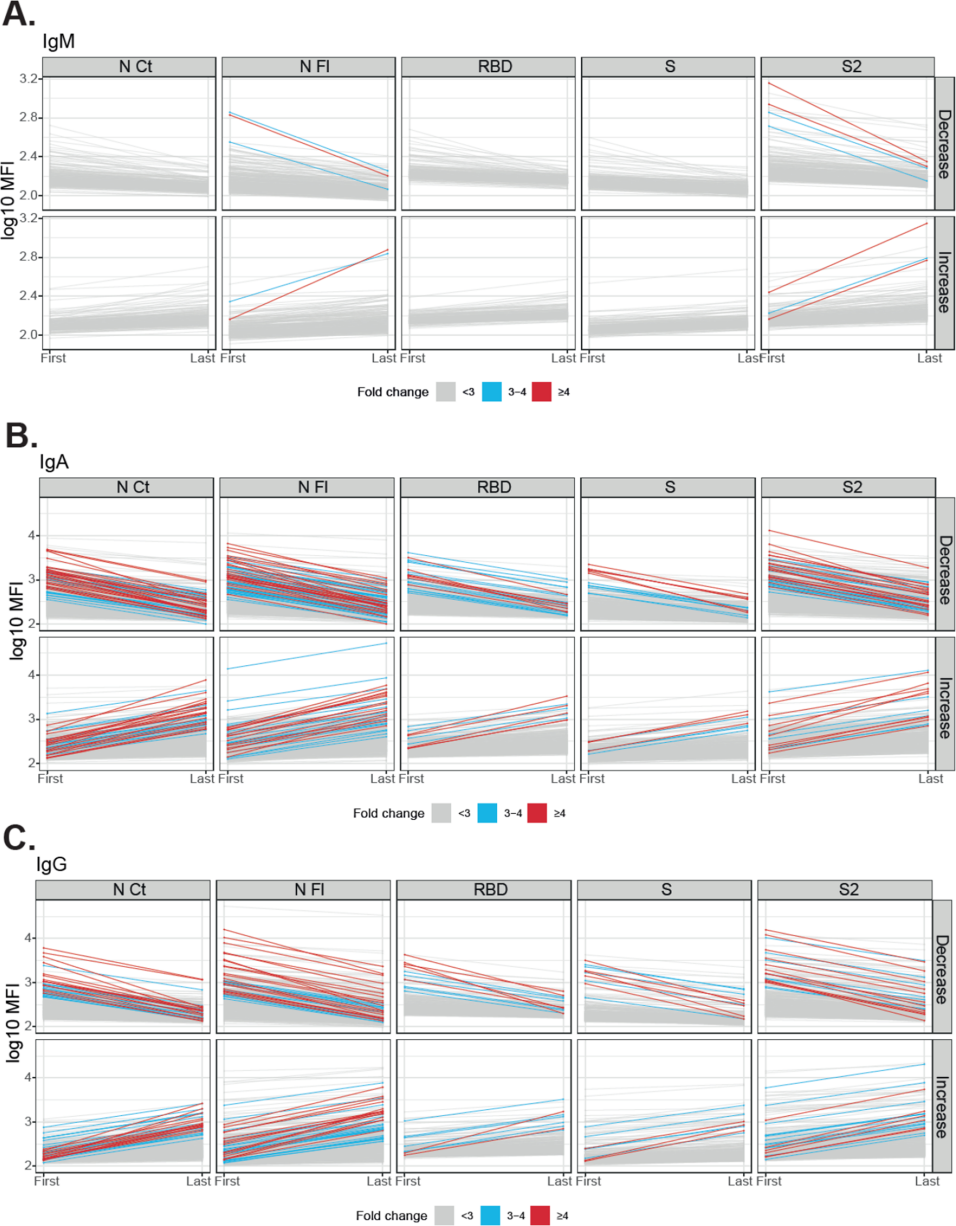
Evolution of IgM, IgA, and IgG levels to SARS-CoV-2 antigens between the first and last visit in paired samples. Individuals who decreased or increased IgM (**A**), IgA (**B**), or IgG (**C**) levels per each isotype and antigen are shown in different plots. Gray lines mean < 3-fold-change, blue lines mean 3–4-fold change, and red lines mean ≥4-fold-change. Table 1 indicates the number and proportion of individuals in each category. The levels of antibodies in individuals with only one sample are depicted in Figure S3

For the estimation of antibody conversion/reversion rates, only data from participants with minimum two visits and minimum 6 days in between visits were considered (1548 participants). Figure 1 and Additional file 3: Figure S1 show the subjects in whom saliva antibody levels to SARS-CoV-2 changed ≥ 3–4-fold from the first to the last visit. Computing those with a ≥ 4-fold increase in antibody levels to at least one Ig/antigen pair, the overall antibody conversion rate was 3.22% (49/1518) (Table 2). This represented a 6 times higher estimate of new SARS-CoV-2 infections than what RT-PCR detected in this subgroup (8/1518, 0.53%). Stratifying by age, IgG conversion rates were significantly higher in adults (2.94%, 11/374) than in children (1.31%, 15/1144) (*p*=0.035) (Additional file 4: Table S2). Antibody conversion was higher for IgA (2.37%, 36/1518) and IgG (1.71%, 26/1518) than for IgM (0.2%, 3/1518). The N FL and N CT proteins were the main targets of saliva antibodies, followed by the S2 protein. Excluding individuals who only increased SARS-CoV-2 N FL antibodies (0.86%, 13/1518), potentially cross-reactive with HCoV N FL [26], the final adjusted conversion rate was 2.36%. Antibodies were maintained at a wide range of levels in a large number of subjects, and in others, they decayed from the first to the last visit (Table 2, Fig. 1), but no one reverted for all isotypes/antigens (≥ 4-fold decrease). SARS-CoV-2 IgAs reverted more than IgGs. Additional file 5: Figure S2 shows the antibody levels in those diagnosed as RT-PCR positive and Additional file 6: Figure S3 shows all subjects, including those with only one sample collected.

**Table 2:** Saliva antibody conversion rates between the first and last study visit

Antibody	Antigen	Increased^a^ *N* (%)			Decreased^b^ *N* (%)			Maintained^c^ *N* (%)
Fold change:		< 4	**≥4**	Total	< 4	≥4	Total	-
**IgM**	**N CT**^d^	207 (13.6%)	0 **(0%)**	207	245 (16.1%)	0 (0%)	245	1066 (70.2%)
	**N FL**	241 (15.9%)	1 **(0.06%)**	242	344 (22.6%)	1 (0.06%)	345	931 (61.3%)
	**RBD**	192 (12.6%)	0 **(0%)**	192	212 (13.9%)	0 (0%)	212	1114 (73.4%)
	**S**	223 (14.7%)	0 **(0%)**	223	243 (16.0%)	0 (0%)	243	1052 (69.3%)
	**S2**	268 (17.6%)	2 **(0.1%)**	270	340 (22.4%)	2 (0.13%)	342	906 (59.7%)
	***Global***		3 **(0.2%)**			0 (0.0%)		-
***N FL only***			1 **(0.1%)**			-		-
**IgA**	**N CT**	524 (34.5%)	20 **(1.3%)**	544	619 (40.8%)	27 (1.7%)	646	328 (21.6%)
	**N FL**	546 (35.9%)	15 **(1.0%)**	561	656 (43.2%)	26 (1.7%)	682	275 (18.1%)
	**RBD**	461 (30.4%)	5 **(0.3%)**	466	551 (36.3%)	7 (0.4%)	558	494 (32.5%)
	**S**	442 (29.1%)	3 **(0.2%)**	445	529 (34.8%)	6 (0.4%)	535	538 (35.4%)
	**S2**	481 (31.7%	8 **(0.5%)**	489	627 (41.3%)	19 (1.2%)	646	383 (25.2%)
	***Global***		36 **(2.3%)**			1 (0.1%)		-
***N FL only***			8 **(0.5%)**			-		-
**IgG**	**N CT**	566 (37.3%)	14 **(0.9%)**	580	509 (33.5%)	14 (1.0%)	523	415 (27.3%)
	**N FL**	586 (38.6%)	15 **(1.0%)**	601	561 (36.9%)	18 (1.3%)	579	338 (22.3%)
	**RBD**	439 (28.9%	2 **(0.1%)**	441	419 (27.6%)	5 (0.3%)	424	653 (43.0%)
	**S**	418 (27.5%)	3 **(0.2%)**	421	376 (24.8%)	5 (0.3%)	381	716 (47.2%)
	**S2**	504 (33.2%)	6 **(0.4%)**	510	500 (32.9%)	12 (0.8%)	512	496 (32.7%)
	***Global***		26 **(1.7%)**			4 (0.3%)		-
***N FL only***			9 **(0.6%)**			-		
**Total**			49 **(3.2%)**			0 (0.0%)		-
***N FL only***			13 **(0.9%)**			-		-

^a^The number (*N*) of subjects who increased antibody levels was calculated for each isotype/antigen pair, per Ig isotype, and globally, out of the 1518 individuals in whom two samples were available with ≥6 days of difference (see also Fig. 1). Individuals who increased antibody levels ≥4-fold change (FC) for at least one isotype/antigen were considered antibody positive. The total saliva antibody conversion rate (% in bold) was calculated as the proportion of positive individuals

^b^A decrease in antibody levels ≥4 FC was interpreted as negativization for any given isotype/antigen pair. Within an individual, complete antibody reversion was considered only if the antibody levels decreased ≥4 FC for all the isotype/antigen pairs

^c^Individuals who maintained antibody levels between visits are computed for comparison

^d^*N* nucleocapsid, *CT* C-terminus end, *FL* full-length, *RBD* receptor binding domain of spike (S). Antibody conversion for N FL is shown separately as representative of potential cross-reactivity with endemic human coronaviruses

### Factors affecting SARS-CoV-2 antibodies

**Fig. 2:**
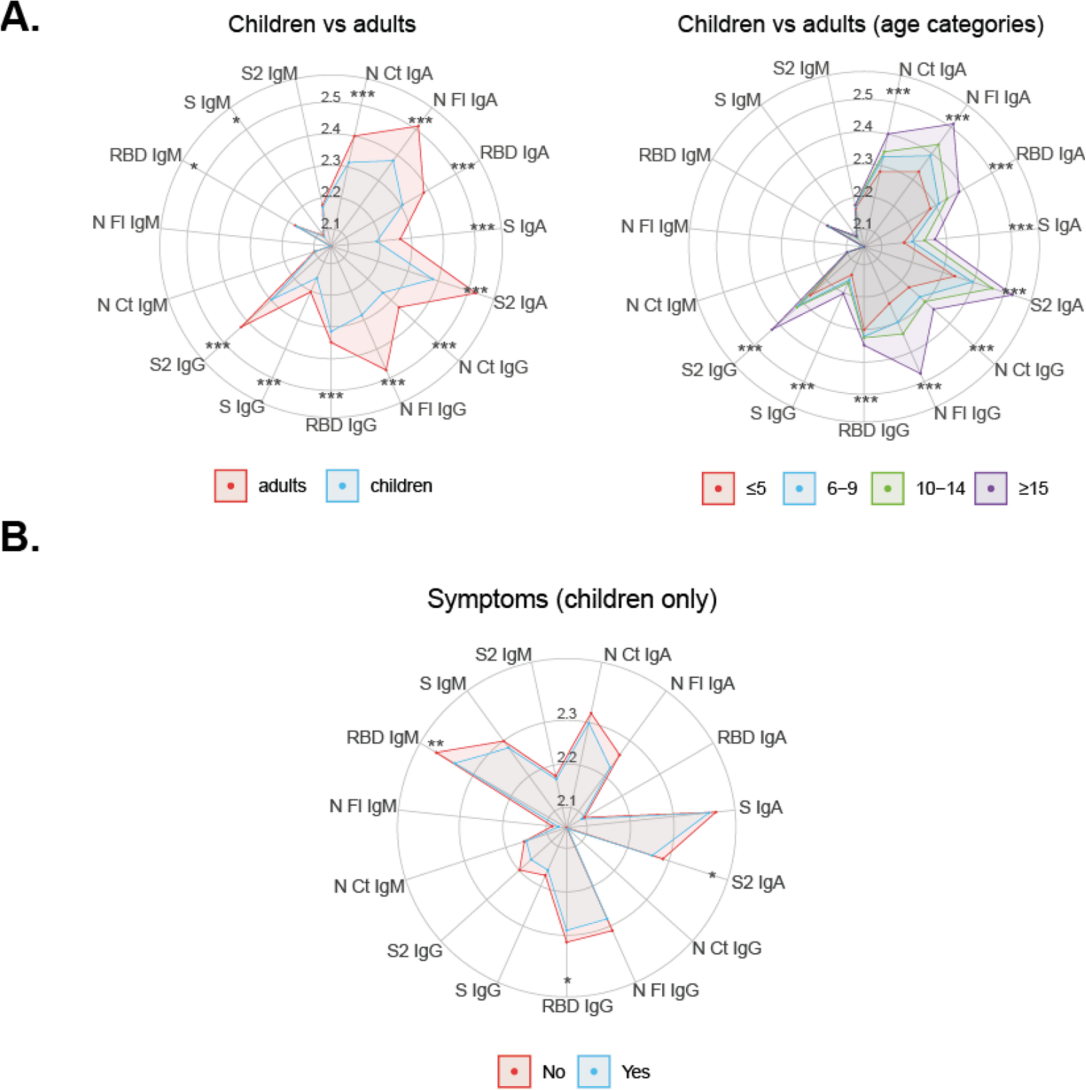
Antibody levels by age and symptoms. Radar charts representing the median of antibody levels (in log_10_MFI) in saliva collected in the last or single visits, comparing children (< 15 years old) versus adults (**A**), and symptomatic (*n*=43, blue) versus asymptomatic (*n*=2635, red) children (**B**). Group medians were compared through Mann-Whitney *U* test. Statistically significant differences between comparisons are highlighted with asterisks. * *p* ≤ 0.05, ** *p* ≤ 0.01, *** *p* ≤ 0.001

Saliva IgA and IgG levels were significantly lower in children (*n*=2677) than in adults (*n*=800), while no differences were seen for IgM (Fig. 2A). IgG and IgA levels gradually increased statistically significantly with age (Additional file 7: Figure S4). Among RT-PCR positives (*n*=10), IgA and IgG levels tended to also be higher in adults compared to children (Additional file 8: Figure S5A). Children with COVID-19-compatible symptoms had statistically significantly lower IgA to S2 and IgM and IgG RBD than children not reporting symptoms (Fig. 2B). Antibody levels were higher in females than males (Additional file 9: Figure S6).

### Multi-marker antibody patterns

**Fig. 3:**
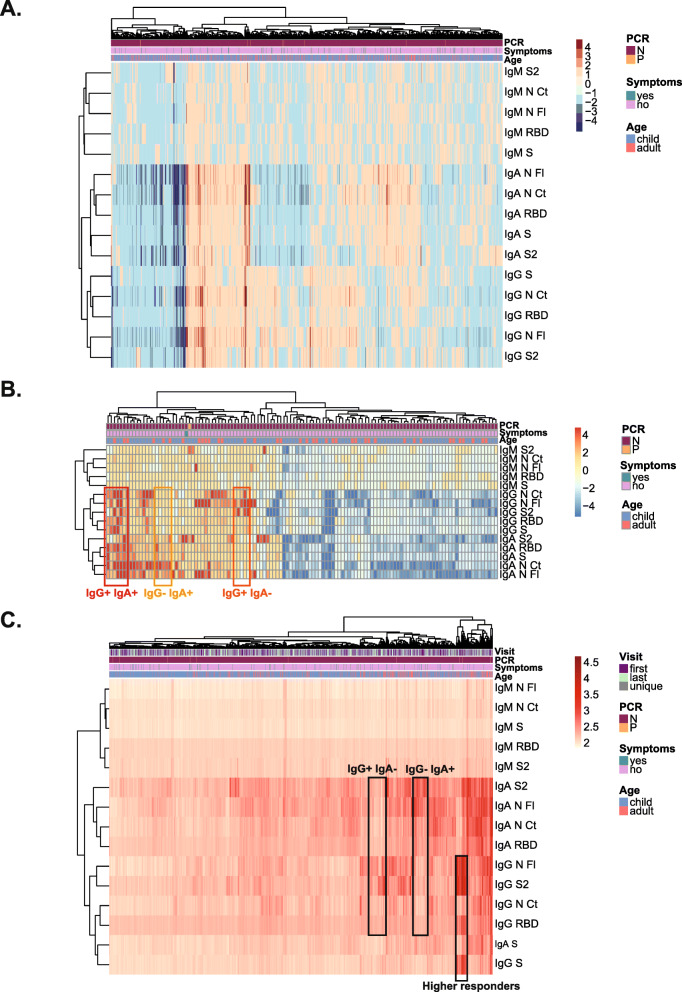
Heatmap analysis of antibody responses per individual. Fold change antibody levels (MFI) with hierarchical clustering (Canberra), including all individuals with paired first and last visit samples, showing decreasers (blue scale), maintainers and increasers (red scale) (**A**) or including only individuals who increased or decreased antibody levels ≥4-fold between the two visits (**B**). Antibody levels (log_10_ MFI) with hierarchical clustering (Euclidean) in all individuals (**C**)

In a heatmap of FC antibodies from the first to the last visit, most individuals with high FC raised both IgG and IgA (very few IgM), and a smaller group showed a raised either IgG or IgA (Fig. 3A). Focusing on individuals with ≥ 4-FC, some increased IgG predominantly, some increased IgA predominantly, and others increased both isotypes (Fig. 3B). There was no clear pattern for age or symptoms.

Combining all antibodies and variables in all individuals, the strongest signal for the high responders mapped to IgG to N FL and S2 antigens, as seen in the antibody conversion analysis, which was accompanied by IgG to S and RBD responses, but lower IgA reactivity (Fig. 3C). Another group of antibody responders had a more predominant IgA than IgG reactivity, while others had a more predominant IgG than IgA reactivity. There were more adults among higher antibody responders and no clear pattern was seen for symptoms.

## Discussion

We showed that a non-invasive screening approach based on weekly saliva sampling in ~ 2000 subjects with thousands of visits, coupled to a high-throughput multiplex assay to quantify antibodies, is capable of measuring infection rates in pediatric populations, being more friendly than serology, which is especially relevant for children. Thus, saliva antibody conversion between two study visits over a 5-week period in our population, based on a ≥ 4-FC increase combining 3 immunoglobulin isotypes and 5 SARS-CoV-2 antigens, was 3.22%, or 2.36% excluding individuals with only N FL antibodies that may cross-react with HCoV [26]. In addition to circumventing the need for blood sampling, saliva surveys are easier to deploy in the field and do not require qualified health care personnel for collection.

Saliva antibody conversion estimates were 6 times higher than the cumulative infection rate derived from weekly RT-PCR screening, despite capturing exposure to the virus with ~ 10–14 days delay in respect to the infection. Of note, 6 out of 8 RT-PCR positive individuals had the viral diagnosis at the final visit, therefore we would not expect antibody conversion in those until some days later.

The finding that a number of potential infections were detected by saliva antibody FC but not by RT-PCR could be related to lower viral loads in asymptomatics and in children [27–29] (the predominant population here), consistent with their lower antibody levels compared to symptomatics and adults. Another explanation is that the virus presence could be more transitory in children [28]. Also, the viral load in saliva lasts shorter than in nasopharyngeal tissue and becomes negative earlier in asymptomatics [30]. Importantly, other studies have also shown that children with a negative RT-PCR can have antibody responses detectable in saliva [31]. Together, data indicate that children can mount an antibody response to SARS-CoV-2 without a viral diagnosis, suggesting that immunity in children could prevent the establishment of SARS-CoV-2 infection.

Interestingly, saliva IgG conversion rates, and levels of IgA and IgG, were significantly lower in children than adults, consistent with different infection and transmission dynamics. A recent study found a negative correlation of age with IgG levels in children and a moderate but positive correlation in adults [32]. The lower levels in children contrast with them being globally more asymptomatic than adults; however, their antibodies could be more efficacious against the virus than in adults

The value of detecting asymptomatic exposure by saliva antibodies may lead to a better determination of the COVID-19 incidence, especially in the school setting. This diagnosis method could allow the saliva self-collection of the children and an easier analysis procedure, to obtain real data about SARS-CoV-2 impact in screening campaigns, for example, leading to better policy decisions with respect to the bubble groups and social distance measures.

Further supporting a role for saliva antibodies on immunity, mucosal IgM, IgA, and IgG to S but not N proteins were significantly higher in individuals not reporting symptoms than in symptomatic ones. This is the opposite of what is commonly observed in blood: symptomatic or severe disease patients have higher viral loads and SARS-CoV-2 antibody levels than asymptomatic individuals, reflecting the intensity of exposure. Our results point to an anti-disease effect of saliva S-specific antibodies that are known to neutralize SARS-CoV-2 invasion via ACE2 receptor in respiratory mucosal tissues. Indeed, there is increasing data on the significant role for mucosal immunity and particularly for secretory as well as circulating IgA antibodies in COVID-19 [33]. Mucosal IgA can have a key role in early SARS-CoV-2-specific neutralizing response [22]. Patients with high saliva viral loads developed antiviral antibodies later than those with lower viral loads [33]. Therefore, studies detecting IgA in addition to IgG in saliva will help to better understand the dynamics of COVID-19 mucosal immunity. Thus, saliva antibody assays could be valuable to monitor vaccine take and correlates of protection when inhaled or intranasal boosters become available [34].

Due to the more transient nature of SARS-CoV-2 antibody responses in oligosymptomatic patients, reliance on measuring serum IgA and IgG might underestimate the percentage of individuals who have experienced COVID-19. In addition to serum, measurement of mucosal IgA should be considered, as local responses may be higher than systemic in such cases, or it could be that the response is only mucosal. IgA in mild COVID-19 cases can often be transiently positive in serum [34], and serum IgG may remain negative or become positive many days after symptom onset, while IgA could appear faster in saliva. Thus, an added benefit of saliva serological surveys is that it may catch people with no or transient IgA or IgG serum responses but detectable IgA levels in nasal fluid [35]. Here, the measurement of both IgG and IgA in saliva increased the probability to identify positive responders because not all subjects produced both isotypes at the time of sampling.

Regarding kinetics, many individuals appeared to maintain antibody levels similar to the ones observed in increasers over the follow-up period, with no reversions. A faster decay in antibodies was seen for IgA than for IgG, consistent with its shorter half-life. Systemic IgG antibodies may be maintained in COVID-19 patients for at least 12 months post symptoms onset [36–38]. Less information is available on the long-term kinetics of mucosal antibodies, which would be relevant to investigate it in follow-up studies.

Levels of saliva antibodies were higher to N than to S antigens. This shows that antibodies to N proteins, not included in current first-generation vaccines, are nevertheless immunogenic and may be useful to track viral exposure in saliva field surveys and after vaccination. There is higher cross-reactivity for N than S antigens among different coronaviruses, and higher levels of pre-existing antibodies to some seasonal HCoV could provide partial immunity against COVID-19 [26, 39].

The main study limitation was the unavailability of pre-pandemic saliva samples that did not allow establishing the positivity threshold by the classical method, but the use of ≥4 FC metric is valid as indicated by WHO and EMA guidelines. A related constraint was that we could not relate saliva antibodies to the current infection because there were very few RT-PCR positives, and that we could not compare saliva to serum responses due to the unavailability of blood samples. However, studies showing a significant correlation between saliva and serum antibody levels [18–20] indicate that our approach could also be applicable to study the persistence of immunity and reinfections following COVID-19 vaccination at a larger scale.

## Conclusion

Antibody profiling in saliva samples with a multiplex technique represents a helpful and simpler tool in community-based surveys for determining saliva antibody conversion and prevalence of SARS-CoV-2 exposure in a school-like environment. Saliva antibodies and conversion in the 2020 initial pandemic waves were lower in children than adults, and levels were higher in asymptomatic than symptomatic individuals, pointing to an anti-disease protective role of mucosal immunoglobulins. This non-invasive screening technique can help study the dynamics of the pandemic and guide policies about maintaining schools and holiday camps active, particularly in later waves when SARS-CoV-2 community transmission could be higher in unvaccinated children, and when the circulation of more contagious variants could make them more vulnerable to disease manifestations. This approach will also be useful to study reinfections over time as well as immunogenicity and persistence of immunity after COVID-19 vaccination at a larger scale, due to the distinct N and S antigen specificities evaluated, particularly when mucosal vaccine boosters become available.

## Supplementary Information

**Additional file 1.** Detailed methods

**Additional file 2: Table S1.** Baseline characteristics

**Additional file 3: Figure S1.** Radar charts of saliva antibodies by visit

**Additional file 4: Table S2.** Fold change antibody levels between first and last visit

**Additional file 5: Figure S2.** Antibody levels from first to last visits and in unique samples in RT-PCR positives

**Additional file 6: Figure S3.** Levels of antibodies at the first, last and single visits

**Additional file 7: Figure S4.** Antibody levels by age

**Additional file 8: Figure S5.** Antibody levels by age and RT-PCR results

**Additional file 9: Figure S6.** Radar charts of antibody levels by sex

## Data Availability

All data are available from the corresponding authors upon request and will be deposited at the Universitat de Barcelona open repository (10.34810/data145).

## Abbreviations

SARS-CoV-2: Severe acute respiratory syndrome coronavirus
COVID-19: Coronavirus disease
HCoV: Common cold human coronaviruses
ACE2: Angiotensin-converting enzyme 2
S: Spike
RBD: Receptor binding domain
FL: Full length
CT: C-terminus
MFI: Median fluorescence intensity
FC: Fold change
BSA: Bovine serum albumin
PBS: Phosphate buffered saline
MFI: Median fluorescence intensity
RT-PCR: Real-time polymerase chain reaction
EMA: European Medicine Agency
WHO: World Health Organization

## References

[1] Brotons P, Launes C, Buetas E, Fumado V, Henares D, de Sevilla MF (2020). Susceptibility to SARS-CoV-2 infection among children and adults: a seroprevalence study of family households in the Barcelona Metropolitan Region, Spain. Clin Infect Dis.

[2] Cruz AT, Zeichner SL (2020). COVID-19 in children: initial characterization of the pediatric disease. Pediatrics..

[3] Lu X, Zhang L, Du H, Zhang J, Li YY, Qu J (2020). SARS-CoV-2 infection in children. New Eng J Med..

[4] Ludvigsson JF (2020). Children are unlikely to be the main drivers of the COVID-19 pandemic - a systematic review. Acta Paediatr..

[5] Bi Q, Wu Y, Mei S, Ye C, Zou X, Zhang Z, Liu X, Wei L, Truelove SA, Zhang T, Gao W, Cheng C, Tang X, Wu X, Wu Y, Sun B, Huang S, Sun Y, Zhang J, Ma T, Lessler J, Feng T (2020). Epidemiology and transmission of COVID-19 in 391 cases and 1286 of their close contacts in Shenzhen, China: a retrospective cohort study. Lancet Infect Dis..

[6] Cao Q, Chen Y-C, Chen C-L, Chiu C-H (2020). SARS-CoV-2 infection in children: Transmission dynamics and clinical characteristics. J Formos Med Assoc..

[7] Kelvin AA, Halperin S (2020). COVID-19 in children: the link in the transmission chain. Lancet Infect Dis..

[8] Liguoro I, Pilotto C, Bonanni M, Ferrari ME, Pusiol A, Nocerino A, Vidal E, Cogo P (2020). SARS-COV-2 infection in children and newborns: a systematic review. Eur J Pediatr..

[9] Mallapaty S (2020). How do children spread the coronavirus? The science still isn’t clear. Nature..

[10] Davies NG, Klepac P, Liu Y, Prem K, Jit M, Pearson CAB (2020). Age-dependent effects in the transmission and control of COVID-19 epidemics. Nat Med..

[11] Khan T, Rahman M, Al AF, SSY H, Ata M, Zhang Q (2021). Distinct antibody repertoires against endemic human coronaviruses in children and adults. JCI Insight.

[12] Nogrady B. How kids’ immune systems can evade COVID. Nature. 2020;588:382. 10.1038/d41586-020-03496-7 33303982

[13] Pavel AB, Wu J, Renert-Yuval Y, Del Duca E, Glickman JW, Miller RL, et al. SARS-CoV-2 receptor ACE2 protein expression in serum is significantly associated with age. Allergy. 2020;76(3):875–878. 10.1111/all.14522. 10.1111/all.14522 PMC8278339 32726474

[14] Esteve-Sole A, Anton J, Pino-Ramírez RM, Sanchez-Manubens J, Fumadó V, Fortuny C (2021). Similarities and differences between the immunopathogenesis of COVID-19-related pediatric inflammatory multisystem syndrome and Kawasaki disease. J Clin Invest..

[15] Yüce M, Filiztekin E, Özkaya KG (2021). COVID-19 diagnosis -a review of current methods. Biosens Bioelectron..

[16] Péré H, Podglajen I, Wack M, Flamarion E, Mirault T, Goudot G, Hauw-Berlemont C, le L, Caudron E, Carrabin S, Rodary J, Ribeyre T, Bélec L, Veyer D (2020). Nasal swab sampling for SARS-CoV-2: a convenient alternative in times of nasopharyngeal swab shortage. McAdam AJ, editor. J Clin Microbiol..

[17] Teo AKJ, Choudhury Y, Tan IB, Cher CY, Chew SH, Wan ZY, Cheng LTE, Oon LLE, Tan MH, Chan KS, Hsu LY (2021). Saliva is more sensitive than nasopharyngeal or nasal swabs for diagnosis of asymptomatic and mild COVID-19 infection. Sci Rep..

[18] Griffin SM, Converse RR, Leon JS, Wade TJ, Jiang X, Moe CL, Egorov AI (2015). Application of salivary antibody immunoassays for the detection of incident infections with Norwalk virus in a group of volunteers. J Immunol Methods..

[19] Wade TJ, Griffin SM, Egorov AI, Sams E, Hudgens E, Augustine S, DeFlorio-Barker S, Plunkett T, Dufour AP, Styles JN, Oshima K (2019). Application of a multiplex salivary immunoassay to detect sporadic incident norovirus infections. Sci Rep..

[20] Pisanic N, Randad PR, Kruczynski K, Manabe YC, Thomas D, Pekosz A, et al. COVID-19 serology at population scale: SARS-CoV-2-specific antibody responses in saliva. J Clin Microbilology. 2021;59(1):e02204–20. 10.1128/JCM.02204-20. 10.1128/JCM.02204-20 PMC7771435 33067270

[21] Dobaño C, Vidal M, Santano R, Jiménez A, Chi J, Barrios D, Ruiz-Olalla G, Rodrigo Melero N, Carolis C, Parras D, Serra P, Martínez de Aguirre P, Carmona-Torre F, Reina G, Santamaria P, Mayor A, García-Basteiro AL, Izquierdo L, Aguilar R, Moncunill G (2020). Highly sensitive and specific multiplex antibody assays to quantify immunoglobulins M, A and G against SARS-CoV-2 antigens. J Clin Microbiol..

[22] Sterlin D, Mathian A, Miyara M, Mohr A, Anna F, Claër L (2021). IgA dominates the early neutralizing antibody response to SARS-CoV-2. Sci Transl Med.

[23] Nishanian P, Aziz N, Chung J, Detels R, Fahey JL (1998). Oral fluids as an alternative to serum for measurement of markers of immune activation. Clin Diagn Lab Immunol..

[24] McKie A, Vyse A, Maple C (2002). Novel methods for the detection of microbial antibodies in oral fluid. Lancet Infect Dis..

[25] Jordan I, de Sevilla MF, Fumado V, Bassat Q, Bonet-Carne E, Fortuny C, et al. Transmission of SARS-CoV-2 infection among children in summer schools applying stringent control measures in Barcelona, Spain. Clin Infect Dis. 2021;ciab227. 10.1093/cid/ciab227. Online ahead of print. 10.1093/cid/ciab227 PMC7989514 33709138

[26] Dobaño C, Santano R, Jiménez A, Vidal M, Chi J, Rodrigo Melero N (2021). Immunogenicity and crossreactivity of antibodies to the nucleocapsid protein of SARS-CoV-2: utility and limitations in seroprevalence and immunity studies. Transl Res.

[27] Marks M, Millat-Martinez P, Ouchi D, Roberts CH, Alemany A, Corbacho-Monné M (2021). Transmission of COVID-19 in 282 clusters in Catalonia, Spain: a cohort study. Lancet Infect Dis..

[28] Bellon M, Baggio S, Bausch FJ, Spechbach H, Salamun J, Genecand C (2021). SARS-CoV-2 viral load kinetics in symptomatic children, adolescents and adults. Clin Infect Dis.

[29] Kociolek LK, Muller WJ, Yee R, Dien Bard J, Brown CA, Revell PA, Wardell H, Savage TJ, Jung S, Dominguez S, Parikh BA, Jerris RC, Kehl SC, Campigotto A, Bender JM, Zheng X, Muscat E, Linam M, Abuogi L, Smith C, Graff K, Hernandez-Leyva A, Williams D, Pollock NR (2020). Comparison of upper respiratory viral load distributions in asymptomatic and symptomatic children diagnosed with SARS-CoV-2 infection in pediatric hospital testing programs. J Clin Microbiol..

[30] Iwasaki S, Fujisawa S, Nakakubo S, Kamada K, Yamashita Y, Fukumoto T, Sato K, Oguri S, Taki K, Senjo H, Sugita J, Hayasaka K, Konno S, Nishida M, Teshima T (2020). Comparison of SARS-CoV-2 detection in nasopharyngeal swab and saliva. J Infect..

[31] Tosif S, Neeland MR, Sutton P, Licciardi PV, Sarkar S, Selva KJ, Do LAH, Donato C, Quan Toh Z, Higgins R, van de Sandt C, Lemke MM, Lee CY, Shoffner SK, Flanagan KL, Arnold KB, Mordant FL, Mulholland K, Bines J, Dohle K, Pellicci DG, Curtis N, McNab S, Steer A, Saffery R, Subbarao K, Chung AW, Kedzierska K, Burgner DP, Crawford NW (2020). Immune responses to SARS-CoV-2 in three children of parents with symptomatic COVID-19. Nat Commun..

[32] Yang HS, Costa V, Racine-Brzostek SE, Acker KP, Yee J, Chen Z, Karbaschi M, Zuk R, Rand S, Sukhu A, Klasse PJ, Cushing MM, Chadburn A, Zhao Z (2021). Association of Age With SARS-CoV-2 Antibody Response. JAMA..

[33] Russell MW, Moldoveanu Z, Ogra PL, Mestecky J (2020). Mucosal immunity in COVID-19: a neglected but critical aspect of SARS-CoV-2 infection. Front Immunol..

[34] Sheikh-Mohamed S, Isho B, Chao GYC, Zuo M, Nahass GR, Salomon-Shulman RE, et al. A mucosal antibody response is induced by intra-muscular SARS-CoV-2 mRNA vaccination. MedRxiv. 10.1101/2021.08.01.21261297.

[35] Cervia C, Nilsson J, Zurbuchen Y, Valaperti A, Schreiner J, Wolfensberger A (2021). Systemic and mucosal antibody secretion specific to SARS-CoV-2 during mild versus severe COVID-19. J Allergy Clin Immunol.

[36] Isho B, Abe KT, Zuo M, Jamal AJ, Rathod B, Wang JH (2020). Persistence of serum and saliva antibody responses to SARS-CoV-2 spike antigens in COVID-19 patients. Sci Immunol.

[37] Dan JM, Mateus J, Kato Y, Hastie KM, Yu ED, Faliti CE (2021). Immunological memory to SARS-CoV-2 assessed for up to eight months after infection. Science.

[38] Dobaño C, Ramirez A, Alonso S, Vidal-Alaball J, Ruiz-Olalla G, Vidal M (2021). Persistence and baseline determinants of seropositivity in health care workers up to nine months after COVID-19. BMC Med..

[39] Ortega N, Ribes M, Vidal M, Rubio R, Aguilar R, Williams S, Barrios D, Alonso S, Hernández-Luis P, Mitchell RA, Jairoce C, Cruz A, Jimenez A, Santano R, Méndez S, Lamoglia M, Rosell N, Llupià A, Puyol L, Chi J, Melero NR, Parras D, Serra P, Pradenas E, Trinité B, Blanco J, Mayor A, Barroso S, Varela P, Vilella A, Trilla A, Santamaria P, Carolis C, Tortajada M, Izquierdo L, Angulo A, Engel P, García-Basteiro AL, Moncunill G, Dobaño C (2021). Seven-month kinetics of SARS-CoV-2 antibodies and protective role of pre-existing antibodies to seasonal human coronaviruses on COVID-19. Nat Commun..

