# Supplementary material for: Antibody conversion rates to SARS-CoV-2 in saliva from children attending summer schools in Barcelona, Spain
Source: BMC Med. 2021 Nov 23;19:309. doi: 10.1186/s12916-021-02184-1 (PMC8608564; doi:10.1186/s12916-021-02184-1)
Supplement: Supplementary file 1 — Additional file 1. Detailed methods [file 12916_2021_2184_MOESM1_ESM.docx]

**Additional file 1: Detailed methods**

**RT-PCR diagnosis**

Saliva collected for SARS-CoV-2 RNA detection was introduced into micronic tubes for pathogen inactivation (Zymo DNA/RNA Shield Lysis Buffer™; Zymo Research, Freiburg, Germany). Tubes were processed using a TecanEvo 200 automated liquid handling system. RNA was extracted using the Quick-DNA/RNA Viral MagBead kit (Zymo Research) that was fully automated on a TecanDreamPrep NAP Workstation (Tecan Trading AG, Switzerland). The RT-PCR assays were conducted according to CDC-006-00019 CDC/DDID/NCIRD/ Division of Viral Diseases protocol released on 3/30/2020 and available at https://www.fda.gov/media/134922/download, which included the CDC-approved primers and probes for SARS-CoV-2 N1 or N2 genes and RNaseP human gene as internal control (1,2). Primers and probes were purchased from IDT integrated technologies (qPCR probes - 2019-nCoV CDC EUA Kit). RT-PCR assays were performed at the Centre for Genomic Regulation (CRG) in Barcelona, Spain, and results were validated by a Clinical Microbiologist of Hospital Sant Joan de Déu.

**Measurement of antibodies and data analysis**

SARS-CoV-2 target antigens assays to measure IgG, IgA and IgM included the nucleocapsid (N) full-length (FL) and C-terminus (amino acid residues 340-416, CT), the spike (S) FL produced at CRG, S2 purchased from SinoBiological, and RBD donated by F. Krammer (Mount Sinai, NY). For the quantitative suspension array technology (qSAT) assay, protein-coupled magnetic microspheres (Luminex Corporation, Austin, TX) were added to a 384-well μClear^®^ flat bottom plate (Greiner Bio-One, Frickenhausen, Germany) in multiplex (2000 microspheres per analyte per well) in a volume of 90 μL of Luminex Buffer (1% BSA, 0.05% Tween 20, 0.05% sodium azide in PBS) using Integra Viaflo semi-automatic device (96/384, 384 channel pipette). Pools of plasmas from adults exposed to SARS-CoV-2 were used as positive controls in 2-fold, 8 serial dilutions starting at 1:12.5. Technical blanks consisting of Luminex Buffer and microspheres without samples were added in 4 wells to detect and adjust for non-specific microsphere signal. Saliva negative controls were not added due to the unavailability of pre-pandemic samples in spite of contacting national biobanks. Ten µL of each dilution of the positive control and test saliva samples were added to the 384-well plate using Assist Plus Integra device with 12 channels Voyager pipette (final saliva dilution of 1:10). Paired samples from the same individual were run on the same plate. Plates were incubated for 1 h at room temperature in agitation (Titramax 1000) at 900 rpm and protected from light. Then, the plates were washed three times with 200 μL/well of PBS-T (0.05% Tween 20 in PBS), using BioTek 405 TS (384-well format). Twenty five μL of goat anti-human IgG-phycoerythrin (PE) (GTIG-001, Moss Bio) diluted 1:400, goat anti-human IgA-PE (GTIA-001, Moss Bio) 1:200, or goat anti-human IgM-PE (GTIM-001, Moss Bio) 1:200 in Luminex buffer were added to each well and incubated for 30 min. Plates were washed and microspheres resuspended with 80 μL of Luminex Buffer, covered with an adhesive film and sonicated 20 seconds on a sonicator bath platform, before acquisition on a Flexmap 3D xMAP® instrument. At least 50 microspheres per analyte per well were acquired. Crude median fluorescent intensities (MFI) and background fluorescence from blank wells were exported for each analyte using the xPONENT software. A 4-fold change increase in antibodies was applied as seroconversion metric, following indications by the World Health Organization (WHO) and the European Medicine Agency (EMA) guidelines for other viruses (3,4) infections and vaccinations, and as used in numerous studies (5–8). All data were managed and analysed using R software v4.0.3 (devtools (9), tidyverse (10), ggplot2 (11) and pheatmap (12) packages).

**REFERENCES**

1. Nagura-Ikeda M, Imai K, Tabata S, Miyoshi K, Murahara N, Mizuno T, et al. Clinical Evaluation of Self-Collected Saliva by Quantitative Reverse Transcription-PCR (RT-qPCR), Direct RT-qPCR, Reverse Transcription-Loop-Mediated Isothermal Amplification, and a Rapid Antigen Test To Diagnose COVID-19. J Clin Microbiol. 2020;58(9):e01438-20.

2. Sabino-Silva R, Jardim ACG, Siqueira WL. Coronavirus COVID-19 impacts to dentistry and potential salivary diagnosis. Clin Oral Inves. 2020;24:1619–21.

3. WHO. Laboratory Testing for Middle East Respiratory Syndrome Coronavirus. Interim Guid Geneva, Switz. 2018;1–8.

4. European Medicines Agency. Guideline on clinical evaluation of vaccines. Comm Hum Med Prod. 2018;1–21.

5. Long Q-X, Liu B-Z, Deng H-J, Wu G-C, Deng K, Chen Y-K, et al. Antibody responses to SARS-CoV-2 in patients with COVID-19. Nat Med. 2020;26:845–848.

6. Trieu M-C, Bansal A, Madsen A, Zhou F, Sævik M, Vahokoski J, et al. SARS-CoV-2-Specific Neutralizing Antibody Responses in Norwegian Health Care Workers After the First Wave of COVID-19 Pandemic: A Prospective Cohort Study. J Infect Dis. 2021;223(4):589–99.

7. Zhu F-C, Guan X-H, Li Y-H, Huang J-Y, Jiang T, Hou L-H, et al. Immunogenicity and safety of a recombinant adenovirus type-5-vectored COVID-19 vaccine in healthy adults aged 18 years or older: a randomised, double-blind, placebo-controlled, phase 2 trial. Lancet (London, England). 2020;396(10249):479–88.

8. Xia S, Zhang Y, Wang Y, Wang H, Yang Y, Gao GF, et al. Safety and immunogenicity of an inactivated SARS-CoV-2 vaccine, BBIBP-CorV: a randomised, double-blind, placebo-controlled, phase 1/2 trial. Lancet Infect Dis. 2021;21(1):39–51.

9. Wickham H, Hester J, Chang W. Devtools: Tools to Make Developing R Packages Easier. 2020. p. R package version 2.3.2. https://CRAN.R-project.or.

10. Wickham H, Averick M, Bryan J, Chang W, McGowan L, François R, et al. Welcome to the Tidyverse. J Open Source Softw. 2019;4(43):1686. https://doi.org/10.21105/joss.01686.

11. Wickham H. ggplot2: Elegant Graphics for Data Analysis [Internet]. Springer-Verlag New York; 2016. Available from: https://ggplot2.tidyverse.org

12. Kolde R. Package ‘pheatmap’: pretty heat map. 2019;1-8. https://doi.org/10.21105/joss.01686.
