# Supplementary material for: Antibody conversion rates to SARS-CoV-2 in saliva from children attending summer schools in Barcelona, Spain
Source: BMC Med. 2021 Nov 23;19:309. doi: 10.1186/s12916-021-02184-1 (PMC8608564; doi:10.1186/s12916-021-02184-1)
Supplement: Supplementary file 2 — Additional file 2: Table S1. Baseline characteristics [file 12916_2021_2184_MOESM2_ESM.docx]

**Additional file 2: Table S1.** **Baseline characteristics.**

| **Variable** | **All children** | **Child index cases** | **Adult index cases** |
| --- | --- | --- | --- |
| **Sex, male (n %)** | 827 (54.8) | 16 (53.3) | 2 (33.3) |
| **Age, years** | 8.5 (5-10.5) |  |  |
| **Symptomatic, yes (n%)** | 15 | 15 (50.0) | 3 (50) |
| **Ethnicity** |  |  |  |
| Caucasian | 980(65.1) | 15 (51.7) | 5 (83.3) |
| Asian | 47 (3.1) | 0 (0) | 1 (1.67) |
| Arabian | 117 (7.8) | 2 (6.9) | 0 (0) |
| Gipsy | 7 (0.5) | 0 (0) | 0 (0) |
| Latin-Americans | 225 (14.9) | 9 (31) | 0 (0) |
| Sub-Saharans  Others | 4 (0.3)  0 (0) | 3 (10.3)  - | 0 (0)  - |
| **Drug intake in preceding 30 days** | 145 (9.6) | 19 (63.3) | 2 (33.3) |
| Antibiotics | 11 (0.7) | 2 (6.6) | 1 (1.67) |
| Corticosteroids | 17 (1.1) | 0 (0) | 0 (0) |
| Antipyretics | 23 (1.5) | 9 (30) | 2 (33.3) |
| Others | 108 (7.2) | 19 (63.3) | 2 (33.3) |
| **Previous disease** | 151 (10.1) | 2 (6.7) | 0 (0) |
| **Hospitalization during 2020** | 39 (2.6) | 0 (0) | 0 (0) |
| **Infections in previous 3 months** |  |  |  |
| Respiratory infection | 14 (0.9) | 1 (3.3) | 1 (16.7) |
| Gastroenteritis | 28 (1.9) | 2 (6.9) | 0 (0) |
| Other | 47 (3.1) | 0 (0) | 0 (0) |
