# Supplementary material for: Antibody conversion rates to SARS-CoV-2 in saliva from children attending summer schools in Barcelona, Spain
Source: BMC Med. 2021 Nov 23;19:309. doi: 10.1186/s12916-021-02184-1 (PMC8608564; doi:10.1186/s12916-021-02184-1)
Supplement: Supplementary file 3 — Additional file 3: Figure S1. Radar charts of saliva antibodies by visit [file 12916_2021_2184_MOESM3_ESM.docx]

**Additional file 3: Figure S1. Radar charts of saliva antibodies by visit.** Overall median antibody levels in the first and last visit **(A)**, in the last visit comparing individuals who increased, decreased (≥3 fold-change [FC]) or maintained (<3 FC) responses **(B)**, in the first versus the last visit in individuals who decreased **(C)** or increased **(D)** responses. FC in median antibody levels between first and last visit **(E)**. Groups were compared through Mann-Whitney test. * p ≤ 0.05, ** p ≤ 0.01, *** p ≤ 0.001.


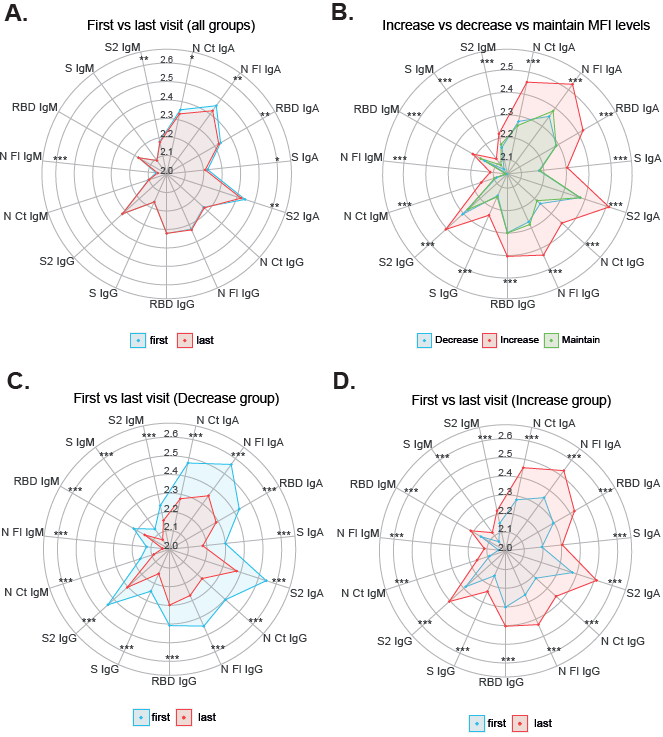

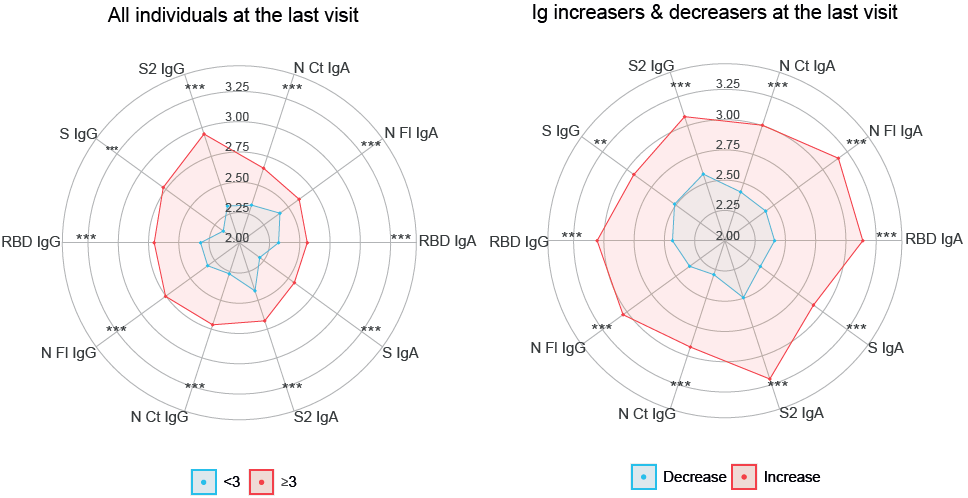


**E.**
