# Supplementary material for: Antibody conversion rates to SARS-CoV-2 in saliva from children attending summer schools in Barcelona, Spain
Source: BMC Med. 2021 Nov 23;19:309. doi: 10.1186/s12916-021-02184-1 (PMC8608564; doi:10.1186/s12916-021-02184-1)
Supplement: Supplementary file 4 — Additional file 4: Table S2. Fold change antibody levels between first and last visit [file 12916_2021_2184_MOESM4_ESM.docx]

**Additional file 4: Table S2.** **Fold change antibody levels between first and last visit stratified by age.** The total number of individuals with paired samples and ≥6 days between them was 1,518, with 1,144 children (age < 15 years) and 374 adults. Antibody conversion was calculated for increase fold change (FC) antibody levels ≥ 4 between visits per immunoglobulin isotype, considering any antigen, and globally, considering any isotype (IgM or IgA or IgG). Antibody reversion was calculated considering that all isotype/antigen pairs had to decrease FC antibody levels ≥ 4 between visits. P-values obtained with two-proportions z-test.

| **Isotype** | **Antigen** | **Antibody conversion**  **(FC increase** ≥**4)** | | | **Antibody reversion**  **(FC decrease** ≥**4)** | |  |
| --- | --- | --- | --- | --- | --- | --- | --- |
|  |  | **Children** | **Adults** | **p-value** | **Children** | **Adults** | **p-value** |
| **IgM** | N CT | 0 **(0.0%)** | 0 **(0.0%)** | - | 0 **(0.0%)** | 0 **(0.0%)** | - |
|  | N FL | 1 **(0.08%)** | 0 **(0.0%)** | 0.567 | 1 **(0.1%)** | 0 **(0.0%)** | - |
|  | RBD | 0 **(0.0%)** | 0 **(0.0%)** | - | 0 **(0.0%)** | 0 **(0.0%)** | - |
|  | S | 0 **(0.0%)** | 0 **(0.0%)** | - | 0 **(0.0%)** | 0 **(0.0%)** | - |
|  | S2 | 2 **(0.17%)** | 0 **(0.0%)** | 0.418 | 2 **(0.2%)** | 0 **(0.0%)** | - |
|  | Global | 3 **(0.26%)** | 0 **(0.0%)** | 0.321 | 0 **(0%)** | 0 **(0%)** | - |
| **IgA** | N CT | 13 **(1.1%)** | 7 **(1.9%)** | 0.278 | 20 **(1.8%)** | 7 **(1.9%)** | 0.879 |
|  | N FL | 9 **(0.8%)** | 6 **(1.6%)** | 0.165 | 19 **(1.7%)** | 7 **(1.9%)** | 0.789 |
|  | RBD | 3 **(0.26%)** | 2 **(0.5%)** | 0.424 | 5 **(0.4%)** | 2 **(0.5%)** | 0.811 |
|  | S | 2 **(0.17%)** | 1 **(0.3%)** | 0.726 | 4 **(0.3%)** | 2 **(0.5%)** | 0.622 |
|  | S2 | 6 **(0.5%)** | 2 **(0.5%)** | 0.981 | 11 **(1.0%)** | 8 **(2.1%)** | 0.076 |
|  | Global | 24 **(2.1%)** | 11 **(2.9%)** | 0.345 | 1 **(0.1%)** | 0 **(0.0%)** | 0.566 |
| **IgG** | N CT | 8 **(0.7%)** | 6 **(1.6%)** | 0.112 | 6 **(0.5%)** | 8 **(2.1%)** | 0.004 |
|  | N FL | 9 **(0.8%)** | 6 **(1.6%)** | 0.165 | 11 **(1.0%)** | 7 **(1.9%)** | 0.159 |
|  | RBD | 2 **(0.17%)** | 0 **(0.0%)** | 0.418 | 1 **(0.1%)** | 4 **(1.0%)** | 0.004 |
|  | S | 2 **(0.17%)** | 1 **(0.3%)** | 0.728 | 2 **(0.2%)** | 3 **(0.8%)** | 0.066 |
|  | S2 | 4 **(0.35%)** | 2 **(0.5%)** | 0.620 | 7 **(0.6%)** | 5 **(1.3%)** | 0.170 |
|  | Global | 15 **(1.3%)** | 11 **(2.9%)** | 0.035 | 1 **(0.1%)** | 3 **(0.8%)** | 0.019 |
| **Total** | | 34 **(3.0%)** | 15 **(4.0%)** | 0.323 | 0 **(0%)** | 0 **(0.0%)** | - |

N: nucleocapsid. FL: full-length. CT: C-terminus end. RBD: receptor binding domain of spike (S).
