# Supplementary material for: Antibody conversion rates to SARS-CoV-2 in saliva from children attending summer schools in Barcelona, Spain
Source: BMC Med. 2021 Nov 23;19:309. doi: 10.1186/s12916-021-02184-1 (PMC8608564; doi:10.1186/s12916-021-02184-1)

**Additional file 5: Figure S2.** **Antibody levels from first to last visits and in unique samples in RT-PCR positives**. RT-PCR positive individuals (in orange) had higher geometric mean of IgA and IgG levels for most antigens than RT-PCR negative individuals (in green), but not statistically significant (data not shown).


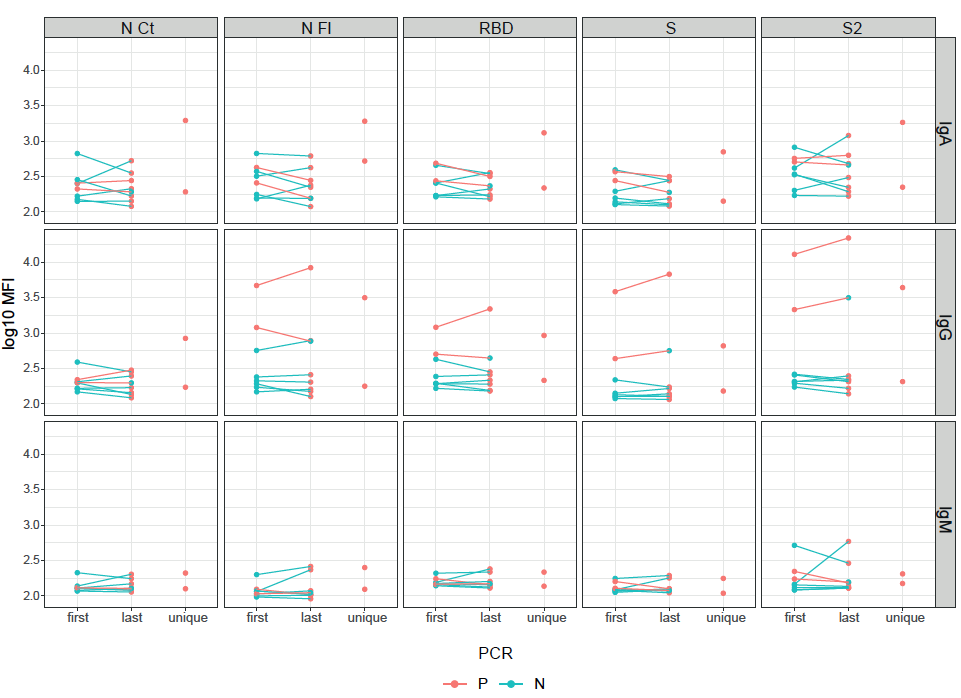

Supplement: Supplementary file 5 — Additional file 5: Figure S2. Antibody levels from first to last visits and in unique samples in RT-PCR positives [file 12916_2021_2184_MOESM5_ESM.docx]
