# Supplementary material for: Antibody conversion rates to SARS-CoV-2 in saliva from children attending summer schools in Barcelona, Spain
Source: BMC Med. 2021 Nov 23;19:309. doi: 10.1186/s12916-021-02184-1 (PMC8608564; doi:10.1186/s12916-021-02184-1)

**Additional file 6: Figure S3. Levels of antibodies at the first, last and single visits**. Groups were compared through Mann-Whitney U test. * p ≤ 0.05, ** p ≤ 0.01, *** p ≤ 0.001. NS = not significant.


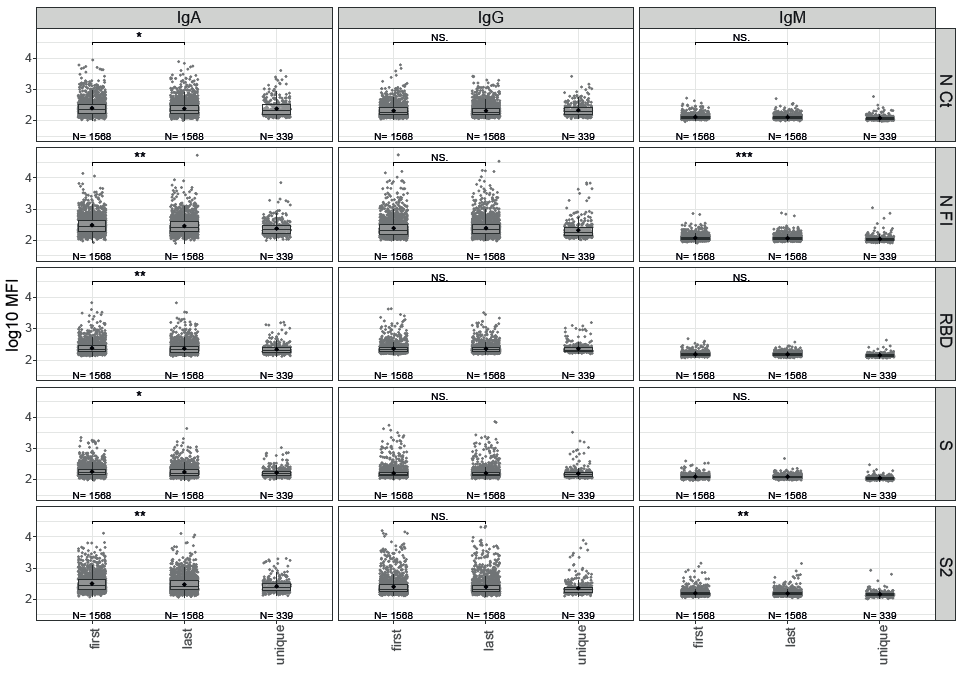

Supplement: Supplementary file 6 — Additional file 6: Figure S3. Levels of antibodies at the first, last and single visits [file 12916_2021_2184_MOESM6_ESM.docx]
