# Supplementary material for: Antibody conversion rates to SARS-CoV-2 in saliva from children attending summer schools in Barcelona, Spain
Source: BMC Med. 2021 Nov 23;19:309. doi: 10.1186/s12916-021-02184-1 (PMC8608564; doi:10.1186/s12916-021-02184-1)

**Additional file 7: Figure S4. Antibody levels by age.** Antibody levels by age category groups in the last and single visits **(A)** and by age and sex **(B)**, compared by Mann-Whitney statistical test, and correlations of antibody levels with age **(C)**. * p ≤ 0.05, ** p ≤ 0.01, *** p ≤ 0.001, NS = not significant.


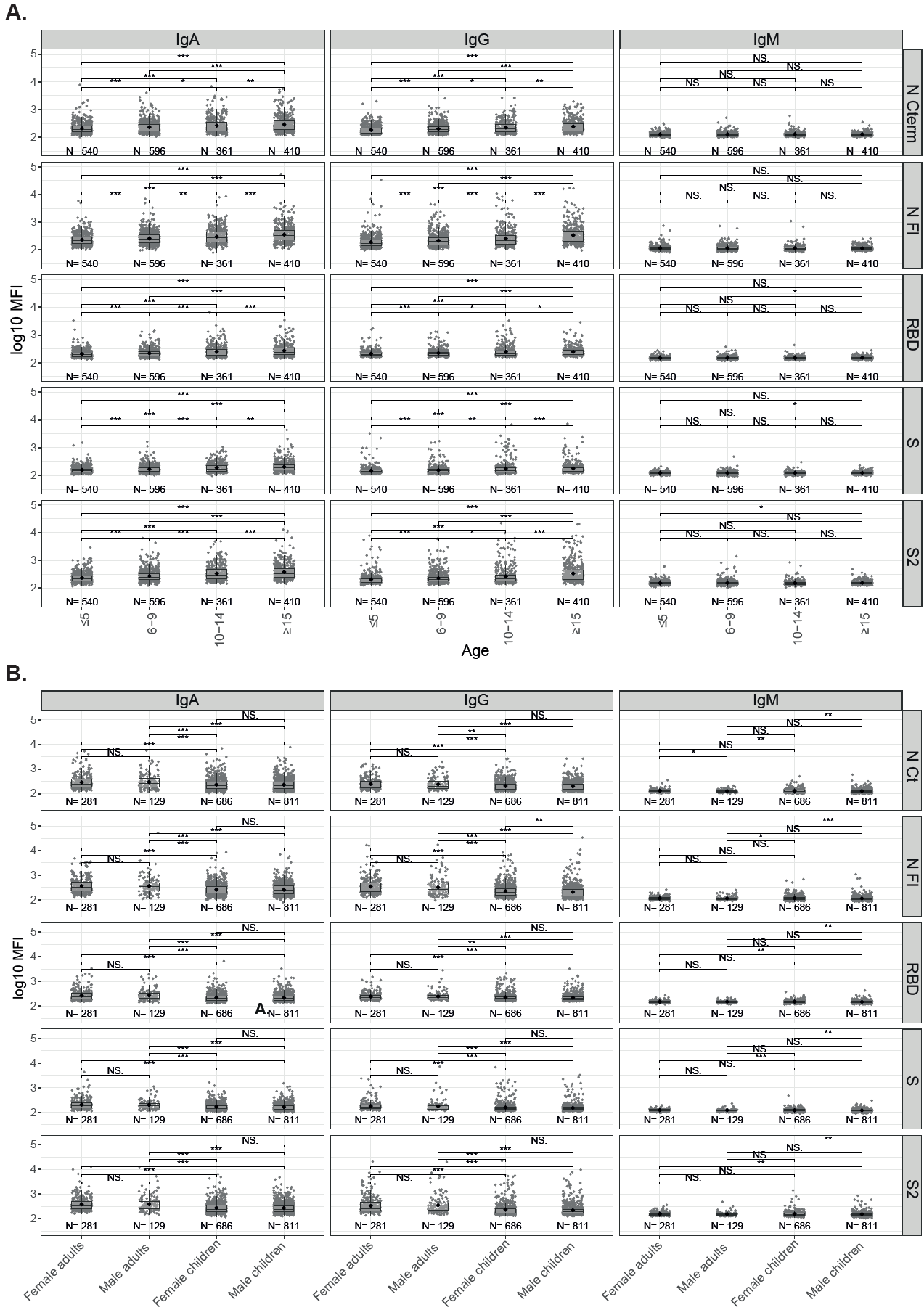


**C.**


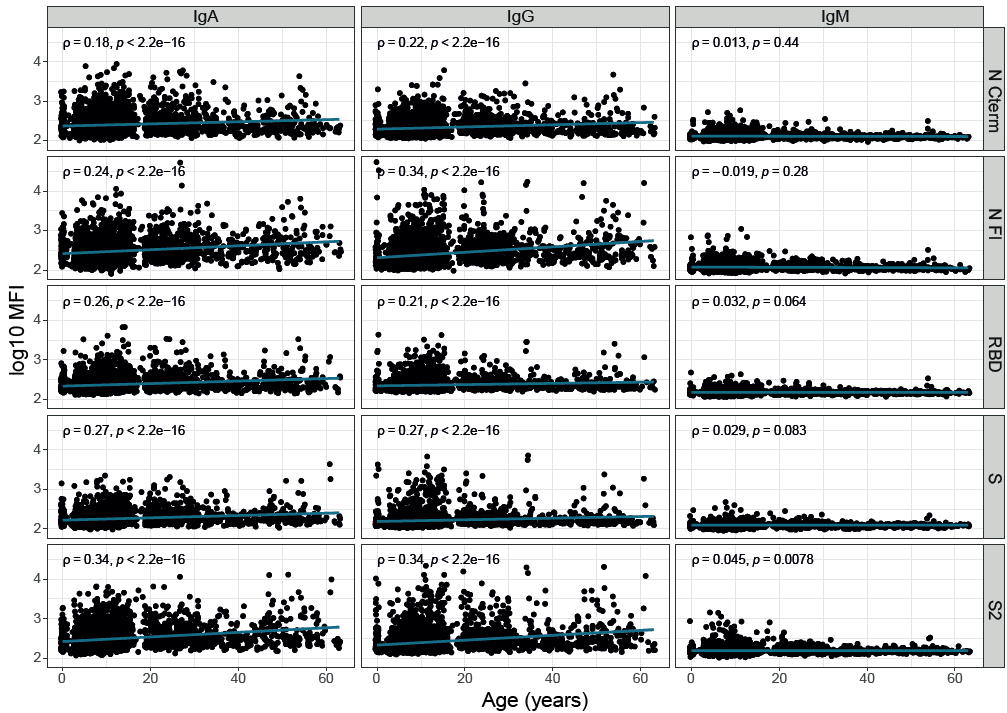

Supplement: Supplementary file 7 — Additional file 7: Figure S4. Antibody levels by age [file 12916_2021_2184_MOESM7_ESM.docx]
