# Supplementary material for: Antibody conversion rates to SARS-CoV-2 in saliva from children attending summer schools in Barcelona, Spain
Source: BMC Med. 2021 Nov 23;19:309. doi: 10.1186/s12916-021-02184-1 (PMC8608564; doi:10.1186/s12916-021-02184-1)
Supplement: Supplementary file 8 — Additional file 8: Figure S5. Antibody levels by age and RT-PCR results [file 12916_2021_2184_MOESM8_ESM.docx]

**Additional file 8: Figure S5. Antibody levels by age and RT-PCR results.** Antibody levels in RT-PCR positive individual by age **(A)** and comparing PCR positive and PCR negative individuals into age groups**(B).**

IgA and IgG levels tended to be higher in adults compared to children (<15 years). Groups were compared by Mann-Whitney U test. NS = not significant.

**
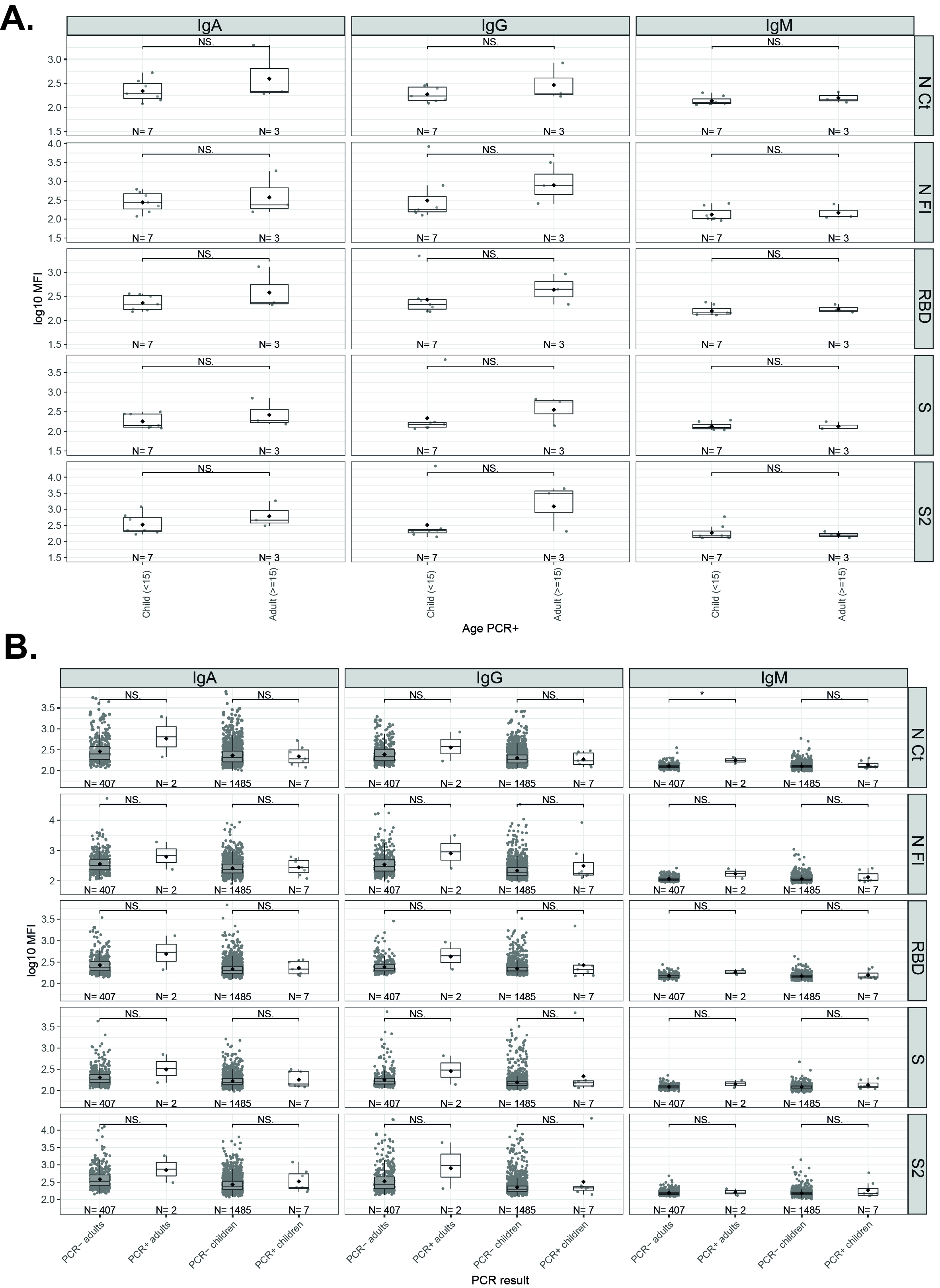
**
