# Supplementary material for: Antibody conversion rates to SARS-CoV-2 in saliva from children attending summer schools in Barcelona, Spain
Source: BMC Med. 2021 Nov 23;19:309. doi: 10.1186/s12916-021-02184-1 (PMC8608564; doi:10.1186/s12916-021-02184-1)

**Additional file 9: Figure S6. Radar charts of antibody levels by sex.** Median antibody levels comparing males (n=940, blue) versus females (n=967, red). Medians were compared through Mann-Whitney U test. * p ≤ 0.05, ** p ≤ 0.01.


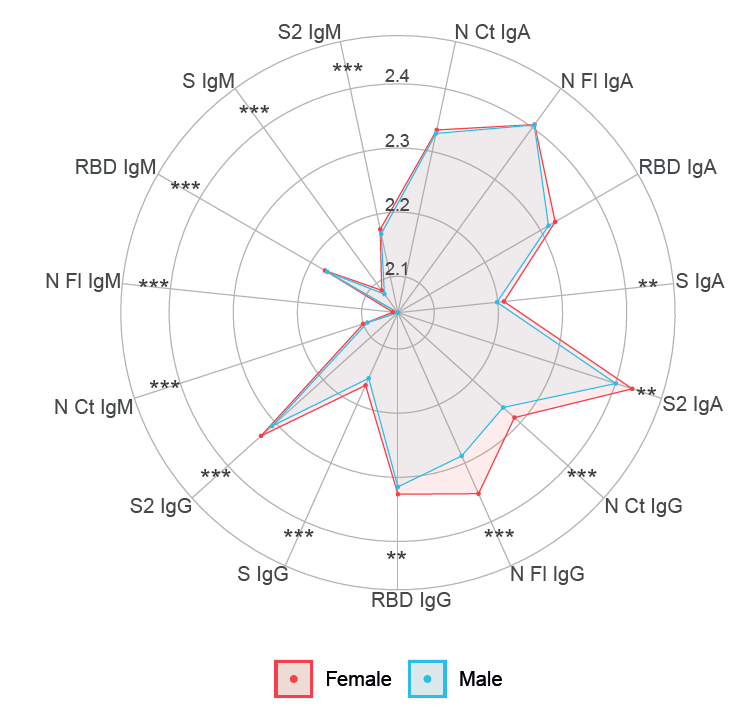

Supplement: Supplementary file 9 — Additional file 9: Figure S6. Radar charts of antibody levels by sex [file 12916_2021_2184_MOESM9_ESM.docx]
